# Abscisic Acid Represses Rice Lamina Joint Inclination by Antagonizing Brassinosteroid Biosynthesis and Signaling

**DOI:** 10.3390/ijms20194908

**Published:** 2019-10-03

**Authors:** Qian-Feng Li, Jun Lu, Yu Zhou, Fan Wu, Hong-Ning Tong, Jin-Dong Wang, Jia-Wen Yu, Chang-Quan Zhang, Xiao-Lei Fan, Qiao-Quan Liu

**Affiliations:** 1 Key Laboratory of Crop Genetics and Physiology of Jiangsu Province/Key Laboratory of Plant Functional Genomics of the Ministry of Education/Jiangsu Key Laboratory of Crop Genomics and Molecular Breeding, College of Agriculture, Yangzhou University, Yangzhou 225009, Chinacqzhang@yzu.edu.cn (C.-Q.Z.); xlfan@yzu.edu.cn (X.-L.F.); 2 Co-Innovation Center for Modern Production Technology of Grain Crops of Jiangsu Province/Joint International Research Laboratory of Agriculture and Agri-Product Safety of the Ministry of Education, Yangzhou University, Yangzhou 225009, China; 3 National Key Facility for Crop Gene Resources and Genetic Improvement, Institute of Crop Sciences, Chinese Academy of Agricultural Sciences, Beijing 100081, China; tonghongning@caas.cn

**Keywords:** abcisic acid, brassinosteroid, hormonal crosstalk, lamina joint inclination, *Oryza sativa* L., RNA-Seq

## Abstract

Leaf angle is a key parameter that determines plant architecture and crop yield. Hormonal crosstalk involving brassinosteroid (BR) plays an essential role in leaf angle regulation in cereals. In this study, we investigated whether abscisic acid (ABA), an important stress-responsive hormone, co-regulates lamina joint inclination together with BR, and, if so, what the underlying mechanism is. Therefore, lamina joint inclination assay and RNA sequencing (RNA-Seq) analysis were performed here. ABA antagonizes the promotive effect of BR on leaf angle. Hundreds of genes responsive to both hormones that are involved in leaf-angle determination were identified by RNA-Seq and the expression of a gene subset was confirmed using quantitative real-time PCR (qRT-PCR). Results from analysis of rice mutants or transgenic lines affected in BR biosynthesis and signaling indicated that ABA antagonizes the effect of BR on lamina joint inclination by targeting the BR biosynthesis gene *D11* and BR signaling genes *GSK2* and *DLT*, thus forming a multi-level regulatory module that controls leaf angle in rice. Taken together, our findings demonstrate that BR and ABA antagonistically regulate lamina joint inclination in rice, thus contributing to the elucidation of the complex hormonal interaction network that optimizes leaf angle in rice.

## 1. Introduction

Leaf angle, defined as the inclination between the leaf blade midrib and the vertical stem of a plant, is an essential component of plant architecture and crop yield [1,2,3,4]. Under good management and appropriate environmental conditions, plants with erect leaves (small leaf angle) show enhanced grain yield per unit area in dense planting populations, due to increased leaf area index (LAI) and photosynthetic efficiency [5,6]. For example, the grain yields of the brassinosteroid (BR)-deficient rice mutant *Osdwarf4-1*, which has erect leaves, is greater in densely planted populations [7]. Therefore, an increasing number of recent studies have focused on identifying the responsible genes/quantitative trait loci (QTLs) that control leaf angles in major crop plants, including rice [8,9,10], maize [11,12,13], and wheat [14,15]. Most recently, two novel QTLs, upright plant architecture1 (*UPA1*) and *UPA2*, have been identified to regulate leaf angle in maize, thus providing the potential to enhance maize yields by increasing planting densities [16].

Leaf angle depends on cell division, expansion, and cell wall composition in the lamina joint [17]. Multiple external and internal factors, including nutrients, carbon dioxide, temperature, phytohormones, and plant genotypes, are involved in modulating leaf angle [10,17,18]. Among these regulators, phytohormone BR is the most important leaf-angle determinant. At present, almost the complete BR signal transduction pathway has been established for the model plant Arabidopsis [19], and several key BR signaling elements have also been well-characterized in rice, includingbrassinosteroid-insensitive1 (*OsBRI1*), glycogen synthase kinase 2/shaggy-like kinase (*OsGSK2*) and dwarf and low-tillering (*DLT*) [20]. OsBRI is localized in the plasma membrane and perceives BR and activates the BR signaling pathway [21]. OsGSK2, the rice ortholog of BR-insensitive 2 (BIN2) in Arabidopsis, functions as the central negative regulator of BR signaling by directly phosphorylating and inactivating downstream transcription factors, including DLT [22]. DLT is a GRAS family protein and is a positive regulator of BR signaling and mediates multiple BR responses in rice [22,23,24]. 

Leaf angle is the most distinctive BR-responsive architectural trait in cereals and ample evidence indicates that BR-deficient or -insensitive mutants exhibit reduced leaf angles in rice and maize [7,16,21,22,25,26]. Conversely, the exogenous application of BR or the genetic enhancement of BR signaling strikingly increased leaf angle [16,22,25]. Therefore, BR might represent a potential biotechnological target with which to enhance crop yield by modulating leaf angle [27]. However, considering the pleiotropic effects of BR on plants, other traits such as seed size and plant height are often closely associated with plant leaf-angle phenotypes [24,28,29]. Thus, attention has focused on identifying novel regulators that interact with the BR pathway and coordinate multiple key cereal agronomic traits, including leaf angle. Among these points of regulation, BR-centered hormonal crosstalk is essential for regulating leaf angle. In rice, both BR and GA promote an increase in leaf angle and the degree of their interaction depends on the respective hormone concentrations [30]. Recently, *OsmiR396d* was implicated in BR–GA co-regulation of leaf angle in rice [31]. The effect of BR and auxin interaction in regulating leaf angle is more complex. Generally, the co-application of BR and auxin has a greater effect in promoting leaf angle than either single hormone treatment [1]. However, rice mutants lacking free auxin have an enhanced BR content and as a consequence, more horizontal leaves [8,32], suggesting that auxin homeostasis is involved in leaf angle regulation. Recently, *TaSPL8*, which encodes a Squamosa-promoter binding protein-like (SPL) protein, was demonstrated to control wheat lamina joint development and leaf angle by modulating auxin signaling and BR biosynthesis [15]. 

Another well-studied phytohormone with an essential role in plant development and adaptation to stress is ABA [33]. In general, BR and ABA have antagonistic regulatory effects. However, the underlying molecular mechanisms have only been recently elucidated by the characterization of BR and ABA signaling pathways. Recently, mechanisms of interaction between BR and ABA have been identified in seed germination [34,35,36], root elongation [37,38,39], stomatal movement [40,41], hypocotyl elongation [42], and drought stress [43]. These represent several nodes of crosstalk and a complex molecular interaction network has been established. However, whether BR and ABA also interact to regulate cereal leaf angles and the underlying molecular mechanism, remains unclear. 

In the present study, lamina joint inclination analysis was applied to evaluate the potential interaction between BR and ABA in controlling leaf angle of rice. A number of rice mutants or transgenic lines affected in BR biosynthesis and signaling were used for further identifying the integration nodes. Furthermore, RNA-seq analysis was accomplished by using the leaf segments to identify genes responsive to both BR and ABA that are involved in leaf-angle determination. Our findings will contribute to the understanding of the complex hormonal interaction network that optimizes leaf angle. 

## 2. Results

### 2.1. ABA Antagonizes the Promotion of Lamina Joint Inclination by BR in Rice

**Figure 1:**
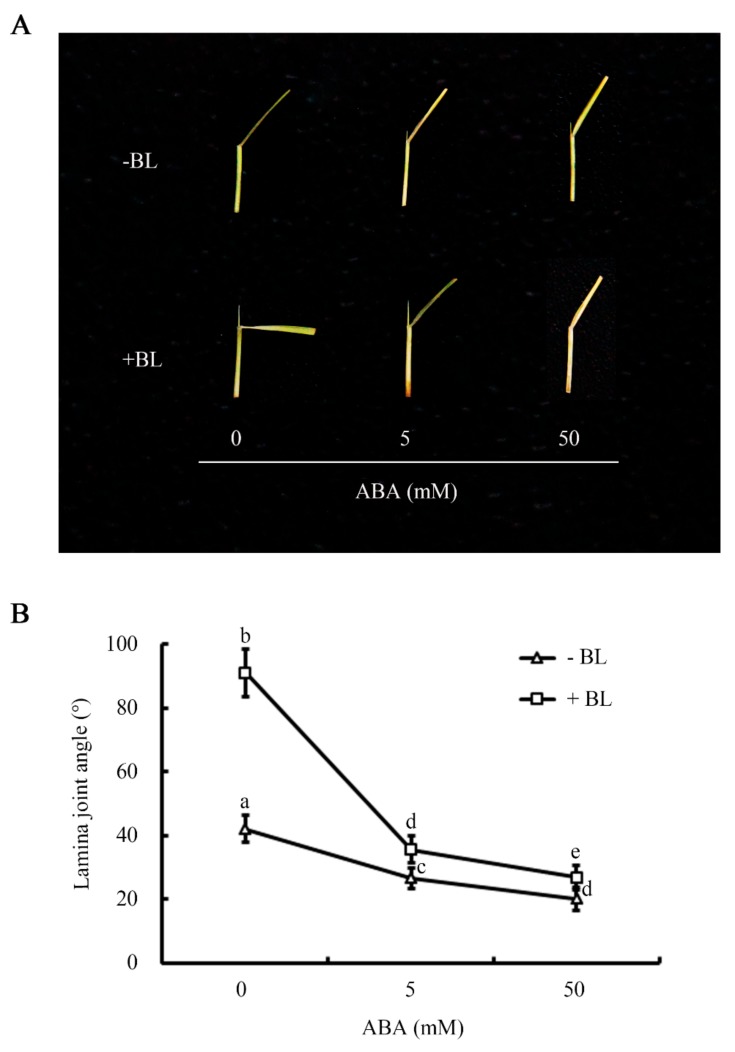
Abscisic acid(ABA) antagonizes the effect of brassinosteroid(BR) on lamina joint inclination in Nipponbare (Nip, *Oryza sativa* L.). (**A**) Responses of leaf segments from etiolated seedlings to different concentrations of ABA in the presence or absence of brassinolide (BL) (10^−7^ M). (**B**) Quantitative data for lamina joint angle analysis. Data were analyzed by analysis of variance (ANOVA) by Pearson’s correction. Error bars represent SD (*n* = 20 seedlings). Bars with different letters indicate statistically significant differences at *p* < 0.05.

The comparison results of lamina joint inclination test showed that the samples exhibited the same leaf-angle responses in both tube-based and Petri-dish-based BL treatment systems, i.e., leaf angle increased together with an increase in BL concentration. However, the leaf-angle response of samples on Petri-dishes was greater than that of samples in tubes (Figure S1A,B). For example, following treatment with 10^−7^ M BL, the mean leaf angle of samples in tubes was 57.2°, whereas that of samples on Petri-dishes was 100.1°. Moreover, the increase in leaf angle was slight at BL concentrations greater than 10^−7^ M in the Petri-dish-based system. Therefore, 10^−7^ M BL treatment on Petri-dishes was chosen for subsequent experiments. To analyze the effect of ABA on leaf angle and to establish a suitable ABA treatment concentration, rice leaf segments were treated with different concentrations of ABA in the presence or absence of 10^−7^ M BL. Leaf angle decreased following treatment with ABA in a concentration-dependent manner. Moreover, when leaf segments were treated with BL and ABA simultaneously, ABA suppressed the positive effect of BL on lamina joint inclination. For example, BL alone resulted in a 91° leaf angle, whereas co-application of 50 mM ABA and 10^−7^ M BL resulted in a 26.9° leaf angle, which was similar to that of samples treated with ABA alone (Figure 1).

### 2.2. Modulating the Expression of BR Biosynthesis Genes Plays an Important Role in ABA Regulation of Lamina Joint Inclination

**Figure 2:**
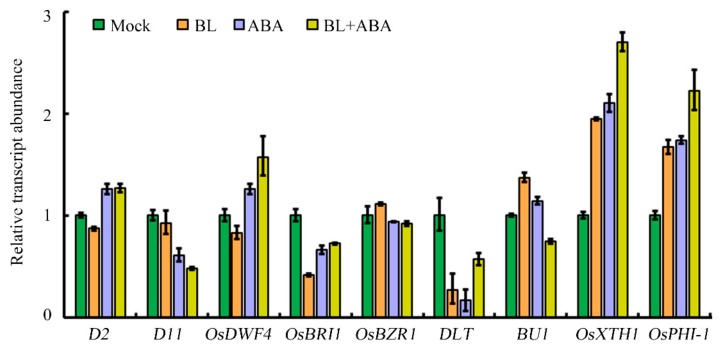
Expression analysis of BR-related genes in Nipponbare treated with BL and/or ABA. *Ubiquitin-conjugating enzyme*(*UBC*) was used as an internal control. Mock refers to dimethylsulfoxide (DMSO) solution. Values were obtained from three independent experiments.

To explore why ABA treatment almost abolished the promotive effect of BR on lamina joint inclination, the expression of several key genes involved in BR biosynthesis, BR signaling, and cell elongation was analyzed. The expression of the three BR biosynthesis genes *ebisu dwarf* (*D2*), *dwarf 11*(*D11*) and *OsDWARF 4* (*OsDWF4*) was slightly suppressed by BL treatment. However, ABA treatment promoted the expression of *D2* and *OsDWF4* but inhibited that of *D11*, independent of the presence of BL (Figure 2). The decrease in expression of *OsBRI1*, which encodes the BR receptor, in response to BL, was partially counteracted by ABA treatment. The genes encoding brassinazole-resistant 1 (OsBZR1) and DLT, two key transcription factors involved in rice BR signaling were differentially affected in response to hormone treatment. Transcription of *OsBZR1* was more or less unaffected by BL or ABA treatment, whereas that of *DLT* decreased markedly. The expression of genes related to cell elongation all increased in response to BL or ABA treatment, although co-application of both hormones slightly reduced the expression of *brassinosteroid upregulated1*(*BU1*) but enhanced the expression of *xyloglucan endotransglycosylase/hydrolase 1* (*OsXTH1*) and *phosphate-induced protein-1* (*OsPHI-1*). Although no consistent changes in expression were observed among the gene classes analyzed, we hypothesized that modulation of *D11* expression is important for the BR–ABA-mediated regulation of leaf angle. The reasons for this are that *D11* was most highly expressed in leaf segments (Figure S2) and the change in *D11* expression in response to BL and ABA was consistent with the results of the lamina joint inclination assay, i.e., ABA suppressed leaf angle and *D11* expression, even in the presence of BL. 

### 2.3. Interaction between ABA and BR Biosynthesis Pathways in Lamina Joint Inclination

**Figure 3:**
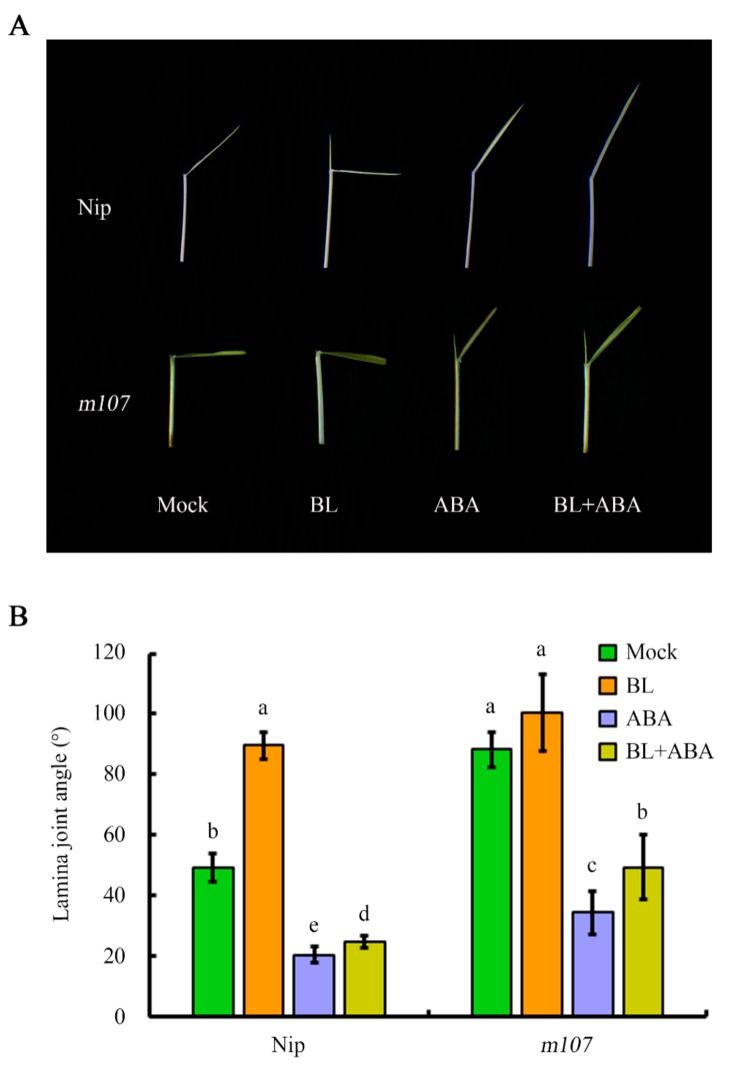
Antagonistic effect of ABA on BR biosynthesis in the regulation of lamina joint inclination. (**A**) Effect of ABA and BR on leaf angle in *m107* mutant and wild type Nip (*Oryza sativa* L.). (**B**) Quantitative data for lamina joint angle analysis in (**A**). Data were analyzed by analysis of variance (ANOVA) with Pearson’s correction. Error bars represent SD (*n* = 20 seedlings). Bars with different letters indicate statistically significant differences at *p* < 0.05.

The most distinctive feature of *m107* mutant is its large leaf angle during the mature stage, making it an ideal genetic background to assess the contribution of *D11* and BR biosynthesis in regulating leaf angle together with ABA. In the absence of hormone treatment, the leaf angle of the *m107* mutant was 88.2°, which was greater than that of the Nip control (49.1°) in the Petri-dish-based experimental system. Furthermore, the leaf angle of mock-treated *m107* was similar to that of BL-treated Nip, suggesting that the overexpression of *D11* due to the dominant mutation indeed enhanced endogenous BR biosynthesis, resulting in a similar effect to exogenous BL treatment. Although ABA treatment still caused a sharp reduction in the *m107* leaf angle, it remained significantly larger than that of wild-type (WT) in the same treatment conditions (Figure 3A,B), indicating that modulating BR biosynthesis contributes to the ABA-mediated regulation of lamina joint inclination.

### 2.4. Identification of the Genes Commonly Responsive to BR and ABA via RNA-Seq

**Figure 4:**
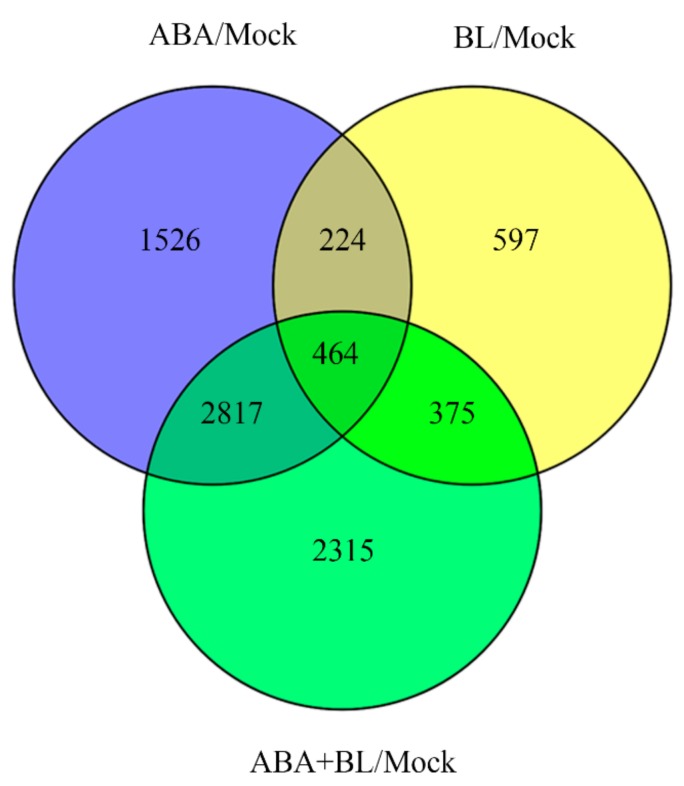
Venn diagram illustrating the number of common target genes in response to ABA, BL, and their combination.

**Figure 5:**
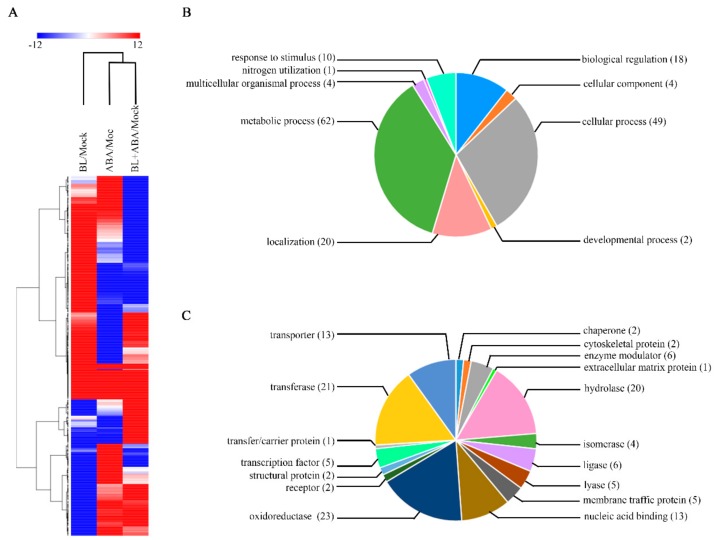
Hierarchical cluster analysis and functional analysis of common differentially expressed genes (DEGs) in three different treatment conditions. (**A**) Hierarchical cluster analysis of common genes in response to BL and/or ABA. Common responsive genes are presented in biological process (**B**) and protein class (**C**) using gene ontology (GO) analysis.

Phytohormones often converge on a set of common target genes to coordinate plant growth and development [44]. Therefore, the identification of genes co-regulated by BR and ABA contributes to understanding the molecular mechanisms that underlie leaf-angle regulation. We used RNA-Seq to identify differentially expressed genes (DEGs) in response to ABA and/or BL treatment. Treatment with ABA or BL alone or co-treatment with both hormones caused the change in expression of thousands of genes, among which, there were 464 common DEGs to all three experimental sets (Figure 4, Table S2). First, the 464 genes were classified into different cluster groups by hierarchical clustering analysis (Figure 5A), suggesting that they perform different roles in mediating ABA–BR crosstalk in controlling leaf angles. Next, these genes were assigned to nine different groups according to their biological processes (Figure 5B). Among these groups, genes belonging to ‘metabolic process’ and ‘cellular process’ constituted the two largest groups and accounted for 36.5% and 28.8% of total analyzed genes, respectively. Based on the annotations of their encoded proteins, the genes could be classified into 17 subfamilies, among which ‘oxidoreductase’ accounted for the largest proportion (17.6%), followed by ‘transferase’ and ‘hydrolase’, which accounted for 16% and 15.3% of the total analyzed genes, respectively (Figure 5C).

**Figure 6:**
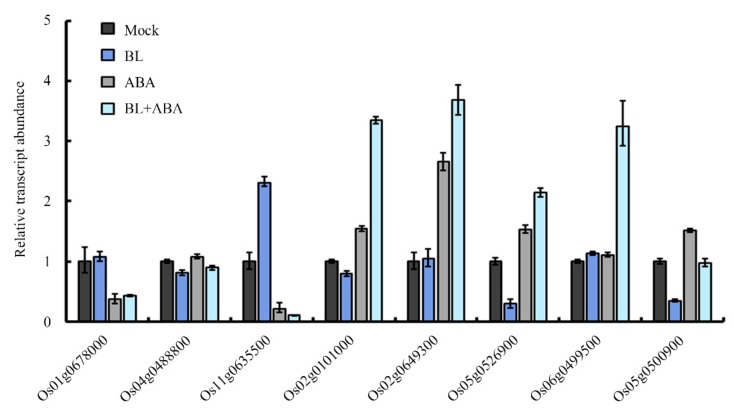
Validation of RNA-Seq data by quantitative real-time polymerase chain reaction (qRT-PCR). *Ubiquitin-conjugating enzyme*(*UBC*) was used as an internal control and values were obtained from three independent experiments.

Next, eight genes out of the 464 common DEGs were randomly selected for qRT-PCR validation. The quantitative expression data confirmed that most selected genes exhibited a similar change in expression to that observed in the RNA-Seq dataset (Figure 6 and Figure S3). For example, the expression of *Os01g0678000* and *Os11g0635500* were induced by BL, but were suppressed by ABA with or without BL, suggesting that the RNA-Seq data are reliable and that many genes are involved in the ABA–BR co-regulation of leaf angle in rice.

### 2.5. Suppression of Lamina Joint Inclination by ABA Does Not Depend on BR Perception

**Figure 7:**
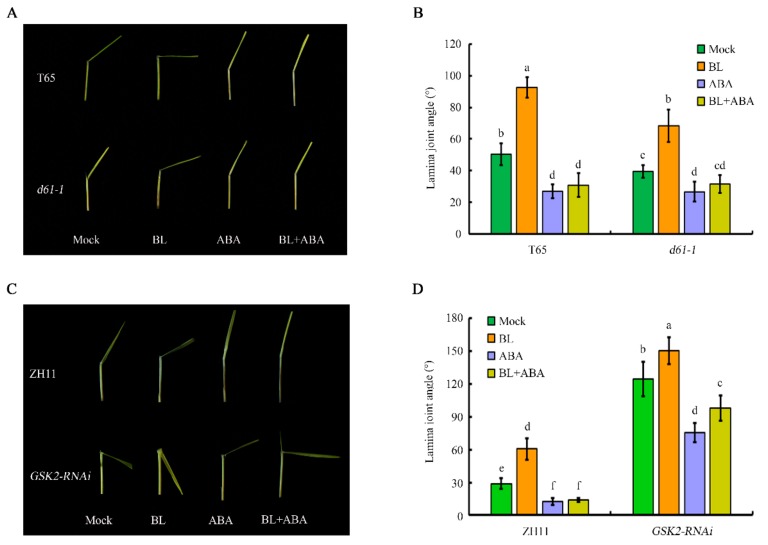
Antagonistic effect of ABA on BR signaling in regulating lamina joint inclination. (**A**) and (**B**) Effect of ABA and BR on leaf angle in the *d61-1* mutant and wild type Taichung 65 (T65, *Oryza sativa* L.). (**C**) and (**D**) Effect of ABA and BR on leaf angle in transgenic rice *GSK2-RNAi* and wild type Zhonghua 11 (ZH11, *Oryza sativa* L.). Data were analyzed by analysis of variance (ANOVA) with Pearson’s correction. Error bars represent SD (*n* = 20 seedlings). Bars with different letters indicate statistically significant differences at *p* < 0.05.

Hormone interactions can exist at the level of biosynthesis or signaling. The data here from exogenous BL treatment and using the *m107* mutant suggested that BR biosynthesis is involved in BR–ABA crosstalk. Lamina joint angles were measured for mutant or transgenic rice lines for genes in the BR signaling pathway, to identify the BR signaling component(s) that mediate ABA–BR crosstalk. We first examined whether a functional BR receptor is required for the reduction in lamina joint inclination caused by ABA. Thus, leaf segments of *d61-1*, a weak allele of *BRI1*, which encodes the BR receptor, were tested for their response to hormone treatment. The leaf angle of *d61-1* was smaller than that of the wild-type under either mock or BL treatments. However, ABA treatment alone or together with BL resulted in a similar leaf angle in *d61-1* and WT (Figure 7A,B), suggesting that the inhibitory effect of ABA on leaf angle resided downstream of the BR receptor. 

### 2.6. BR and ABA Crosstalk in Establishing Lamina Joint Angle Is Partially Mediated by GSK2

**Figure 8:**
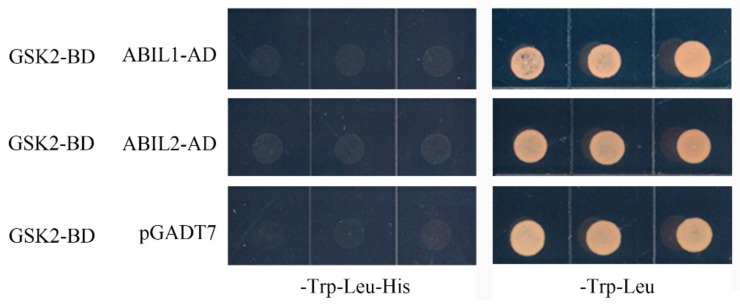
No direct physical interaction was detected between glycogen synthase kinase 2 (GSK2) and ABI-like (ABIL) proteins. Yeast two-hybrid assay for the interaction between GSK2 and ABIL1 and ABIL2. Yeast cells transformed with bait (*pGBKT7-GSK2*) and prey (*ABIL1* or *ABIL2* cloned into *pGADT7*) were selected on SD-Trp/-Leu/-His medium. *pGADT7* was the empty prey vector control.

Because GSK2 is the major negative regulator within the BR signaling pathway, we measured the lamina joint angle of *GSK2-RNAi* transgenic rice, which was 124.6° without BL treatment and was greater than that of the ZH11 control, which was only 28.7° for the same treatment. BL treatment further increased the leaf angle of *GSK2-RNAi* plants to 150.2° compared to that of the WT control, which was only 60.7°. Treatment with ABA alone or together with BL markedly decreased the leaf angle of WT to less than 15°, but only caused a slight reduction in the leaf angle of *GSK2-RNAi* rice, which was 75.6° following ABA treatment alone and 97.9° following simultaneous ABA and BL treatment (Figure 7C,D). We, therefore, hypothesized that GSK2 kinase partially mediates BR–ABA crosstalk in controlling rice leaf angle. ABI1 and ABI2 are two ABA signaling components, which directly interact with and dephosphorylate BIN2, thus modulating its activity in phosphorylating BES1 in Arabidopsis. To verify whether the ABI1/ABI2–BIN2 interaction module also mediates ABA–BR crosstalk in controlling leaf angle in rice, yeast two-hybrid analysis was performed to examine whether GSK2 can physically interact with ABI-LIKE 1 (ABIL1)/ABIL2, the ABI1/ABI2 homologs in rice. The result indicated no interaction (Figure 8), suggesting that although GSK2 is one determinant of lamina joint angle in rice, it probably does not function via the ABI1/ABI2-BIN2 interaction module.

### 2.7. DLT Represents a Major ABA–BR Crosstalk Node in Coordinating Lamina Joint Inclination

**Figure 9:**
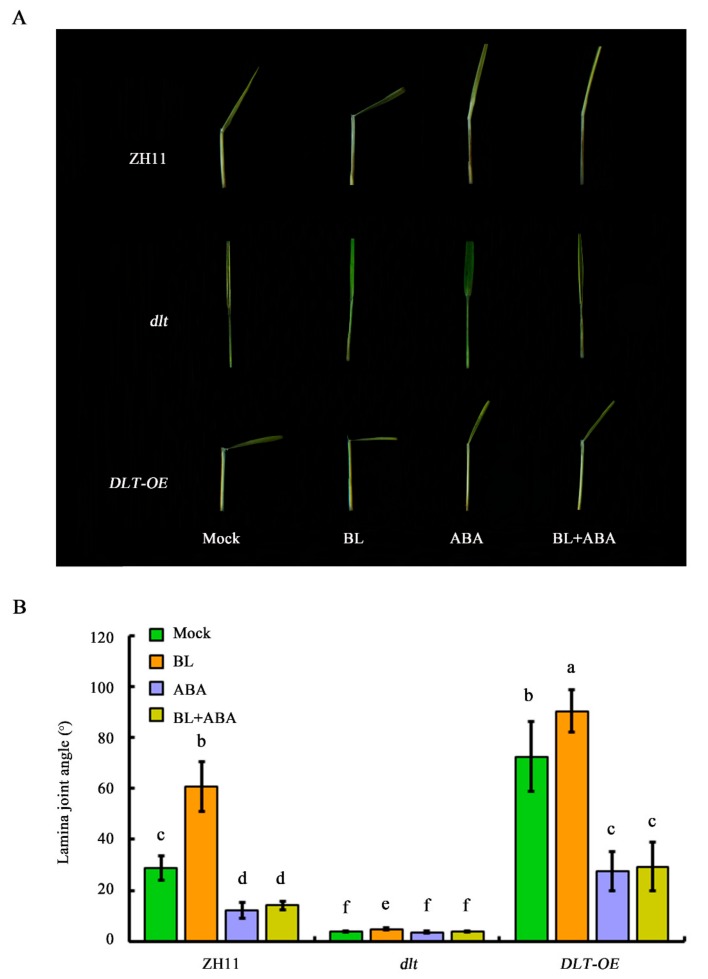
Dwarf and low-tillering(*DLT*) is a major crosstalk node of ABA and BR pathways in coordinating lamina joint inclination. (**A**) and (**B**) Antagonistic effect of ABA and BR on lamina joint inclination in *dlt*, *DLT-OE* transgenic plants and wild type ZH11 (*Oryza sativa* L.). Data were analyzed by analysis of variance (ANOVA) by Pearson’s correction. Error bars represent SD (*n* = 20 seedlings). Bars with different letters indicate statistically significant differences at *p* < 0.05.

The analysis of *GSK2-RNAi* transgenic rice only partially explained the joint regulation of leaf angle by BR and ABA, implying that additional BR signaling component(s) are involved. Because previous reports indicated that DLT is a key regulator of rice lamina joint inclination [22], we tested the response of genetic backgrounds with altered *DLT* expression to BR and/or ABA treatment, to confirm whether *DLT* mediates BR–ABA crosstalk in rice leaf angle regulation. Loss of *DLT* function led to an almost completely erect-leaf phenotype and only BL treatment alone slightly increased the leaf angle. In the presence of BL, ABA application restored the leaf angle to the same as that of the mock-treated control (Figure 9A,B). Moreover, overexpression of *DLT* strikingly increased the leaf angle to 72.4°. Treatment with BL alone increased the leaf angle to 90.3° and ABA treatment decreased the leaf angle to 27.5° and 29.3°, in the presence or absence of BL, respectively. However, the leaf angle of ABA-treated *DLT-OE* rice was still greater than that of the ABA-treated ZH11 control. The response of *DLT-OE* rice in response to BL and/or ABA treatment was qualitatively similar to that of *GSK2-RNAi* rice. However, some differences were observed: Firstly, the leaf angle of *GSK2-RNAi* rice was always greater than that of *DLT-OE* rice in each treatment condition. Secondly, the reduction in the leaf angle of *DLT-OE* rice in response to ABA was greater than that of *GSK2-RNAi* plants. For example, the leaf angles of *GSK2-RNAi* rice were 124.6° and 75.6°, before and after ABA treatment, respectively, but were 72.4° and 27.5°, respectively, for the same treatments with *DLT-OE* rice (Figure 9A,B), representing a more than 60% reduction after ABA treatment. This suggests that *DLT* represents a crosstalk node that integrates BR and ABA signals to modulate rice leaf angle.

## 3. Discussion

The degree of lamina inclination is an important trait that determines crop architecture, photosynthetic efficiency and grain yield [7,17]. Various intrinsic hormonal signals and extrinsic environmental cues are involved in leaf angle regulation and these include BR as a major regulator, particularly in cereals [7,16]. Other phytohormones, including gibberellins, auxin, strigolactone, and JA are also involved in the regulation of leaf angle [1,30,45,46]. However, most of these hormones control leaf angle directly or indirectly by interacting with the BR pathway [1,30,45]. It remains uncertain whether ABA, an important stress-related hormone, also participates in leaf angle regulation and if so, what the underlying molecular mechanism is. In rice, the lamina joint inclination test is one of the most sensitive BR physiological assays and is closely related to leaf angle. Moreover, it is an ideal method with which to investigate the interactions between BR and other hormones in rice. Although evidence supports the existence of crosstalk between BR and ABA in co-regulating seed germination [34,35,36], hypocotyl elongation [42] and stress responses [43], no interaction in modulating rice leaf angle has yet been reported. In this study, evidences indicated that ABA alone can reduce the leaf angle of rice (Figure 1) and co-treatment with ABA and BL strikingly suppressed changes in lamina joint inclination induced by BR (Figure 1). These findings suggest that ABA indeed affects leaf angle in rice, most likely by suppressing BR functions.

BR and ABA are essential for a broad spectrum of plant growth and developmental programs. All hormones, including BR and ABA, control plant development by regulating the expression of a large number of downstream target genes. Therefore, the co-regulation of the same subset of downstream target genes represents one level of hormonal crosstalk [44,47]. Because BR is a major regulator of lamina joint inclination, the expression of several key genes related to BR biosynthesis, signaling, and cell elongation were analyzed by qRT-PCR. The *D11* gene, which encodes the rate-limiting enzyme of BR biosynthesis, is a potential target of both BR and ABA in determining lamina joint inclination. Treatment with ABA alone or with BL reduced *D11* expression. RNA-Seq was performed to identify additional downstream transcripts. A previous study in Arabidopsis revealed more than two hundred overlapping target genes of BL and ABA using a microarray approach [48]. However, a tissue-specific RNA-Seq-based transcriptome analysis, instead of using whole seedlings, can improve spatial resolution and provide more relevant data. In this study, 464 common DEGs were identified in rice leaf segments under three experimental conditions: BL or ABA treatment alone or their co-application. Subsequent qRT-PCR validation showed that for most genes, the change in expression was consistent with that of RNA-Seq data (Figure 6 and Figure S3), confirming the reliability of the transcriptome data. The identification of genes responsive to BL and ABA in the determination of leaf angle is essential for additional functional analyses. Further bioinformatic analysis indicated that more than 60% of these genes were involved in metabolic and cellular processes (Figure 5B). Moreover, nearly half of the genes were oxidoreductases, transferases, and hydrolases (Figure 5C). Several genes involved in phytohormone pathways were also identified. For example, *OsGH3-4* and *OsGH3-7*, two auxin-responsive *GH3* family genes, were regulated by BL and ABA. Another *GH3* family gene, *OsGH3-5*, also integrates auxin and BR signaling to regulate rice leaf angle [8]. Therefore, the transcriptome data here implicate a role for additional genes in the complex regulatory network involving BL and ABA in coordinating rice lamina joint inclination.

In the absence of hormone treatment, the leaf angle of *m107*, the dominant mutant of *D11*, increased, similar to that of WT treated with exogenous BL. Following ABA treatment, the leaf angle decreased greatly, even in the presence of BL treatment (Figure 3A,B). However, the leaf angle of *m107* was still significantly greater than that of WT under the same treatment conditions, suggesting that enhanced BR biosynthesis can partially attenuate the effect of ABA. Consistent with this, expression analysis indicated that ABA led to a decrease in *D11* expression (Figure 2). Similar studies were also performed using several genetic backgrounds with modified BR perception or signaling, including the BR receptor mutant *d61-1*, an RNAi transgenic line of the negative regulator OsGSK2 and a mutant and overexpression line of the transcription factor DLT. These data indicated that integration of the BR and ABA pathways occurs downstream of the BRI receptor, since there was no difference in leaf angle between *d61-1* and the WT in response to ABA (Figure 7). However, knocking down *OsGSK2* expression led to a striking increase in the leaf angle in all hormone treatments. Although ABA treatment caused a decrease in the leaf angle to about 50%, the leaf angle of *GSK2-RNAi* plants was greater than that of the BL-treated WT control (Figure 7), indicating that *GSK2* represents a major node of crosstalk between BR and ABA in leaf angle regulation. Recently, increasing evidence has demonstrated the crucial roles of BIN2/GSK2 in mediating interactions between BR signaling and other pathways in drought responses [43,49], hypocotyl elongation [42,50], seed size [51,52] and cellulose synthesis [53]. Therefore, BIN2/GSK2 might also integrate BR signaling with other pathways, in addition to BZR1/BES1 transcription factors [19]. Furthermore, the DLT transcription factor that functions downstream of GSK2 mediates several BR responses in rice [22,23,24,51]. Most importantly, DLT is involved in BR-induced rice lamina joint inclination: the leaf angle in the *dlt* mutant was relatively small and was hardly affected by BL treatment (Figure 9). However, *DLT* overexpression resulted in a larger leaf angle than WT, even in the presence of ABA. Thus, DLT function can attenuate the effect of ABA on lamina joint inclination. 

In summary, we have shown that BR and ABA antagonistically regulate lamina joint inclination in rice. ABA antagonizes the positive effect of BR on a high lamina joint angle via the BR biosynthesis gene *D11* and the BR signaling genes *GSK2* and *DLT*, thus forming a multi-level regulatory module to control rice leaf angle. Our findings contribute to understanding the complex BR–ABA interaction network, which orchestrates functions of BR and ABA in diverse environmental conditions and thus coordinates plant growth and developmental programs.

## 4. Materials and Methods

### 4.1. Plant Materials

In this study, seedlings of rice (*Oryza sativa* L.) were used for the following experiments. In general, three different *Japonica* cultivars, including Nipponbare (Nip), Zhonghua 11 (ZH11), and Taichung 65 (T65), and derivative mutants or transgenic lines were used. In more detail, *m107* is a *D11* dominant mutant caused by the insertion of a double 35S enhancer T-DNA upstream from its start codon [54], and its wild-type counterpart is Nip. The *GSK2-RNAi* and *DLT-OE* transgenic lines and the *dlt* mutant all derived from recipient ZH11. *d61-1* is a BR-insensitive mutant and its wild-type counterpart is T65. All rice plants were grown under the same climate and management conditions during the summer in a paddy field at Yangzhou University. Mature seeds from superior rice spikelets were collected for experiments.

### 4.2. Lamina Joint Inclination Assay

The lamina joint assay was performed according to [55] with some modifications. In brief, mature seeds were sterilized with 70% ethanol and rinsed twice with sterile water. Seeds were imbibed in darkness for two days and synchronously germinating seeds were selected and grown in the dark for a further eight days at 30 °C. Leaf segments, consisting of 1 cm of the second leaf blade, the lamina joint and 1 cm of the leaf sheath, were excised from the uniform seedlings. Leaf segments were then floated on Milli-Q water for 24 h and were dipped into a centrifuge tube or floated on a Petri-dish containing various hormone treatments in the dark for 48 h. Stock solutions of BL, ABA or an equal volume of ethanol were diluted in 2.5 mM maleic acid potassium solution for treatment. The lamina joint angle, formed by the lamina and leaf sheath, was measured using ImageJ software, a free and Java-based image-processing package supported by the National Institute of Health (https://imagej.nih.gov/ij/). Twenty seedlings were used for each treatment and all experiments were repeated at least three times.

### 4.3. Quantitative Real-Time PCR (qRT-PCR) Analysis

Leaf segments treated with either BL (10^-7^ M), ABA (50 mM), both hormones or an ethanol control for 12 h were used for RNA extraction and gene expression analysis. Total RNA was isolated using the RNeasy Plant Mini Kit (Qiagen, Hilden, Germany) and treated with DNase I (Qiagen). RNA quality and quantity were evaluated using a NanoDrop 2000 (Thermo Scientific, MA, USA) system. High-quality RNA samples were reverse-transcribed using the SuperScript First-Strand Synthesis System (Invitrogen, Van Allen Way Carlsbad, CA, USA) and qRT-PCR was performed using SYBR Premix Ex Taq (TaKaRa, Dalian, Liaoning, China) and an ABI PRISMTM 7700 sequence detector system (Applied Biosystems, Foster City, CA, USA). Relative expression was calculated using the 2^−ΔΔCt^ method, and *ubiquitin conjugase* (*UBC*) was used as the internal control for normalization. Primers are listed in Supplementary Materials Table S1.

### 4.4. RNA-Seq Analysis

High-quality RNA was isolated and used for library construction, and high-throughput RNA sequencing was performed on a BGISEQ-500 platform at the Beijing Genomics Institute (BGI, Shenzhen, China). Raw sequencing reads were trimmed and the collected clean data were aligned to the genome of rice japonica cultivar Nipponbare (IRGSP-1.0, http://rapdb.dna.affrc.go.jp/) using TopHat2 software [56]. The DESeq2 (version 1.12.4) was used to determine differentially expressed genes (DEGs) [57], which were selected according to the following default criteria: A fold change ≥ 2.0 and FDR ≤ 0.001.

### 4.5. Bioinformatic Analysisand qRT-PCR Validation of RNA-Seq Data

The DEGs common to all three treatment experimental sets were isolated for the following validation and bioinformatic analysis. First, common DEGs were used to construct a heat map with Morpheus online software (https://software.broadinstitute.org/morpheus), a frequently used -omics method to study genes with similar expression patterns. Gene ontology (GO) was used for the classification of gene function and the description of genes or gene-product attributes. Using the PANTHER database, genes were analyzed with two sets of ontologies, including biological process and protein class [58]. Finally, qRT-PCR was used to validate the expression of eight randomly selected DEGs from the RNA-Seq data.

### 4.6. Yeast Two-Hybrid Assays

The full-length coding sequence of *GSK2* was amplified and cloned into the *pGBKT7* bait vector and transformed into the yeast strain AH109. Yeast cells carrying the bait vector were transformed with the prey plasmids containing the full-length coding sequences of *ABIL1* or *ABIL2*. Transformants were selected on SD dropout plates. The assay was performed according to the manufacturer’s instructions (TaKaRa, Dalian, Liaoning, China).

### 4.7. Statistical Analysis

For sample characterization, at least three independent experiments were performed unless otherwise specified. All data represent the means ± standard deviation (SD) of the replicates. The data were analyzed by analysis of variance (ANOVA) with Pearson’s correction.

## Supplementary Materials

Supplementary materials can be found at https://www.mdpi.com/1422-0067/20/19/4908/s1. Figure S1. Comparison between the Petri-dish-based and tube-based lamina joint inclination assay. Figure S2. Expression analysis of BR-related genes in Nipponbare. Figure S3. Transcript levels of selected target genes from RNA-Seq analysis. Table S1. Primers used for qRT-PCR analysis in this study. Table S2. The 464 common DEGs in response to ABA and/or BL treatment from RNA-seq analysis.

## Abbreviations

ABA	abscisic acid
ABIL1	ABI-like 1
ANOVA	analysis of variance
BIN2	BR-insensitive 2
BL	brassinolide
BR	brassinosteroid
BU1	brassinosteroid upregulated1
BZR1	brassinazole resistant1
D2	ebisud warf
D11	dwarf 11
DEGs	differentially expressed genes
DLT	dwarf and low-tillering
DMSO	dimethylsulfoxide
GO	gene ontology
GSK2	glycogen synthase kinase 2
LAI	leaf area index
OsBRI1	brassinosteroid-insensitive1
OsDWF4	osdwarf 4
OsXTH1	xyloglucan endotransglycosylase/hydrolase 1
PHI-1	phosphate-induced protein-1
qRT-PCR	quantitative real-time polymerase chain reaction
QTLs	quantitative trait loci
RNA-Seq	RNA sequencing
SD	standard deviation
SPL	Squamosa-promoter binding protein-like
UBC	ubiquitin-conjugating enzyme
UPA1	upright plant architecture1
