# Supplementary material for: Abscisic Acid Represses Rice Lamina Joint Inclination by Antagonizing Brassinosteroid Biosynthesis and Signaling
Source: Int J Mol Sci. 2019 Oct 3;20(19):4908. doi: 10.3390/ijms20194908 (PMC6801706; doi:10.3390/ijms20194908)
Supplement: Supplementary file 1 [file ijms-20-04908-s001.zip › ijms-601089 sp original/Supplementary Materials/Figure S1-S3.pdf]

Supplementary figures

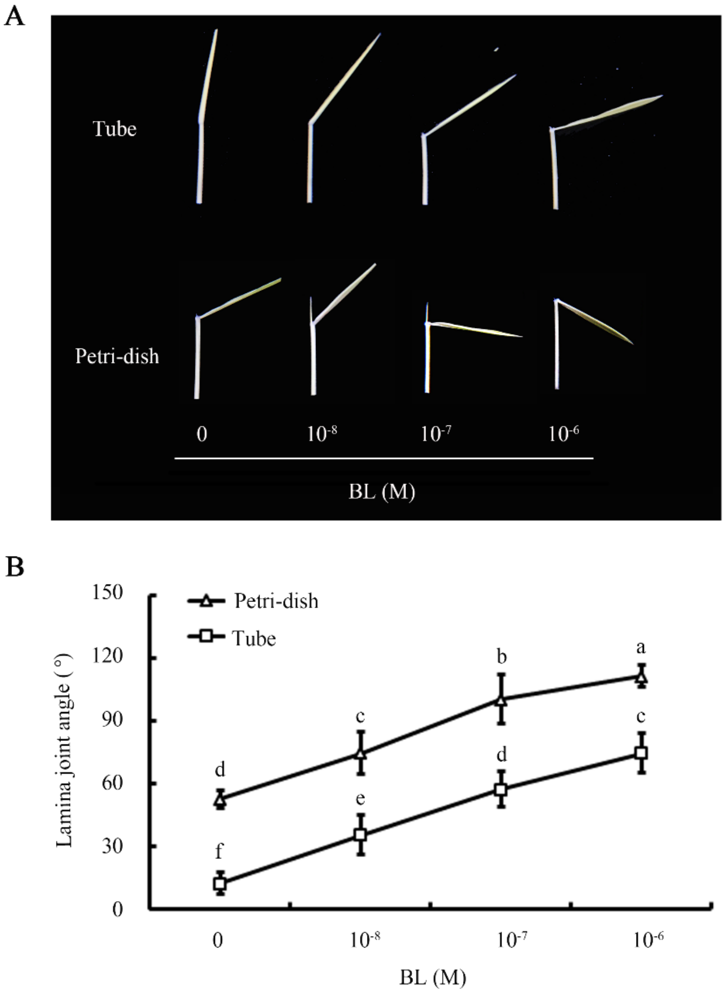

Figure S1. Comparison between petri-dish-based and tube-based lamina joint inclination assay of rice. (A) Effect of different concentrations of BL on lamina joint inclination in tube-based and petri-dish-based treatment conditions. (B) Quantitative data for lamina joint inclination assay as described in (A). Error bars represent SD ($n > 20$ seedlings). Bars with different letters indicate statistically significant differences at $p < 0.05$.

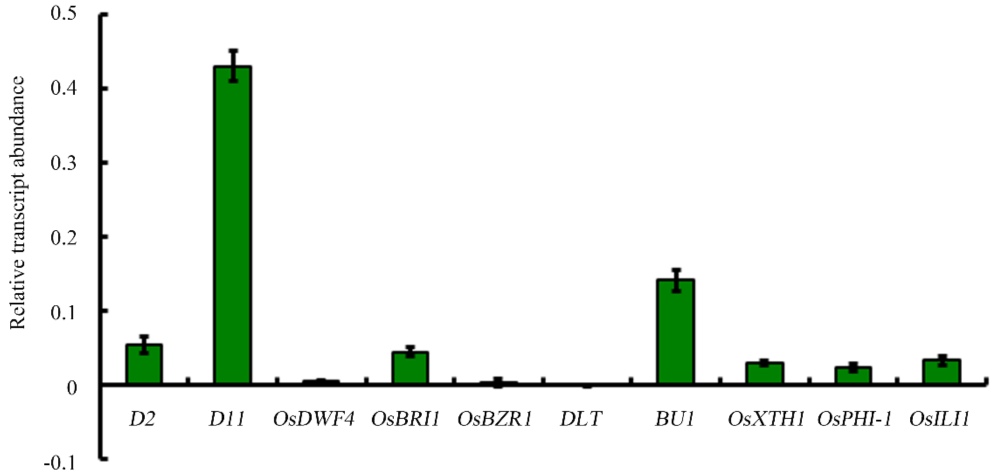

Figure S2. Expression analysis of BR-related genes in Nipponbare. Gene transcript abundance was normalized to that of internal control *UBC* and values were obtained from three independent experiments.

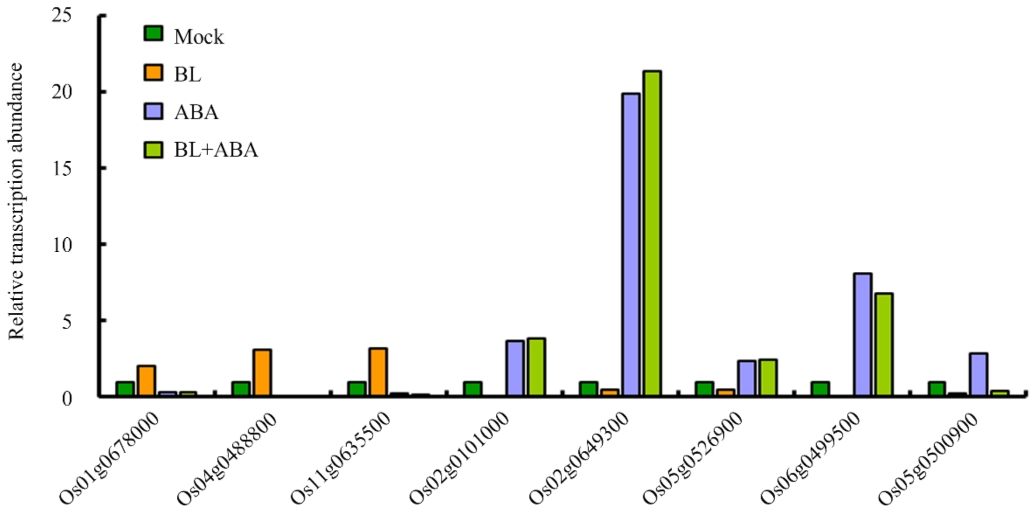

Figure S3. Transcript levels of selected target genes from RNA-seq analysis.
