# Supplementary material for: Abscisic Acid Represses Rice Lamina Joint Inclination by Antagonizing Brassinosteroid Biosynthesis and Signaling
Source: Int J Mol Sci. 2019 Oct 3;20(19):4908. doi: 10.3390/ijms20194908 (PMC6801706; doi:10.3390/ijms20194908)
Supplement: Supplementary file 1 [file ijms-20-04908-s001.zip › ijms-601089 sp original/Supplementary Materials/Table S1.pdf]

Table S1 Primers used for qRT-PCR analysis in this study

Primer Names	Sequence (5' → 3')
D2qRT-F	TCGCTGACGGAGCTGATG
D2qRT-R	ACTTGAGGTGGGAGGACTTG
D11qRT-F	TGGCGACATTGAGAAGATTGC
D11qRT-R	CAGAAGGCGATGACATTGACC
OsDWF4qRT-F	CAGTTTCATGAAGGCCCGGA
OsDWF4qRT-R	GTTCAGCTCCGCCTCCATAC
OsBRI1qRT-F	CAGCTACTTGGCTATCTTGAAGCTCAGC
OsBRI1qRT-R	CCATTCTTGTTGAAGGTGTACTCCGTGC
OsBZR1qRT-F	CGTCGCCCACCTACAACCTC
OsBZR1qRT-R	TCGCCCAAATCGCAGCAT
DLTqRT-F	TGCGGATACTCAACGCCATCA
DLTqRT-R	ACTCGCCGACTCCGGTGATC
BU1qRT-F	GTAGCCAGCTTGATCTCATCTC
BU1qRT-R	GGGACGACTCTACTGCATCA
OsXTH1qRT-F	GATCCGGGAGATGAAGAACCA
OsXTH1qRT-R	TCCATCTGCTGGTCGTACCC
OsPHI1qRT-F	ACGGGAGGAAATACCTGGTC
OsPHI1qRT-R	AATTTTAGCCGCGTGGAAC
Os01g0678000qRT-F	CCGAAAACCAGACCACCAGA
Os01g0678000qRT-R	ACAACTACTGGGCTCTCCCT
Os04g0488800qRT-F	GAAATCGCCTTCTTTGGACGG
Os04g0488800qRT-R	GGAGGCAGGTGGAACTCTG
Os011g0635500qRT-F	CCCAAGAGTGATGCAAAAGGC
Os011g0635500qRT-R	ATAGTTCCTTGGGGCACATGAA
Os02g0101000qRT-F	TCCTACGCCACCCACCAAT
Os02g0101000qRT-R	GAGGGAAGCAGGTACTGGGA
Os02g0649300qRT-F	ATCACCTAGACTACTTGGGCGG
Os02g0649300qRT-R	TGCTACTGCGTTCCATTCCAC
Os05g0526900qRT-F	TCAGAGGAAGGCTGGCAATG
Os05g0526900qRT-R	CGCTGCAAATCATCCACGAA
Os06g0499500qRT-F	GCCACTTGATCCACCATCCC
Os06g0499500qRT-R	ATGCCGTCCTGAGAAGTGAA
Os05g0500900qRT-F	AGCATCGACTCCGACAAGAC
Os05g0500900qRT-R	GTACACGCTGTTGAGCCCTT
OsUBCqRT-F	CCGTTTGTAGAGCCATAATTGCA
OsUBCqRT-R	AGGTTGCCTGAGTCACAGTTAAGTG
